# Comparison of the effectiveness and safety of vaginal and sublingual low-dose misoprostol versus vaginal dinoprostone for labor ınduction: A retrospective cohort study

**DOI:** 10.1371/journal.pone.0336025

**Published:** 2025-10-31

**Authors:** Elmin Eminov, Ayşe Eminov

**Affiliations:** 1 Department of Obstetrics and Gynecology, Faculty of Medicine, Duzce University, Duzce, Turkiye; 2 Faculty of Health Sciences, Kocaeli Health and Technology University, Kocaeli, Turkiye; JSI, ETHIOPIA

## Abstract

**Background:**

Labor induction is one of the most common obstetric interventions, yet the optimal pharmacological agent and route of administration remain subjects of ongoing debate. Prostaglandin analogs such as misoprostol and dinoprostone are widely used for cervical ripening and induction; however, evidence comparing their effectiveness and safety across different routes is still limited.

**Aim:**

The primary purpose of the study is to evaluate the efficacy and safety of vaginal and sublingual misoprostol and vaginal dinoprostone pessary in labor induction.

**Materials and Methods:**

Patients were divided into three groups based on the labor induction methods used. The first group included patients who received 25 µg vaginal misoprostol for labor induction, the second group included patients who received 25 µg sublingual misoprostol, and the third group included patients who received vaginal dinoprostone pessary. The primary outcome of the study was to evaluate vaginal delivery rates and time from induction to delivery of the fetus. The secondary outcomes of the study were to evaluate cesarean delivery rates, indications for cesarean delivery, first- and fifth-minute Apgar scores, birth weight, gender, amniotic fluid meconium contamination, and admission rates to the neonatal intensive care unit (NICU). The study data were collected from the hospital’s automation system and archives. All patients’ demographic and obstetric characteristics and maternal and fetal outcomes were recorded in a spreadsheet document (Microsoft Excel™), and statistical analyses were performed using the SPSS 28.0 program.

**Results:**

Vaginal delivery rates were higher in the group where vaginal (86,5%) and sublingual misoprostol (85,3%) were applied, and cesarean delivery rates were higher in the group where vaginal dinoprostone pessary (31,6%) was applied (p = 0.000). When the groups were compared according to the duration of labor, no difference was found in the vaginal (523.86 ± 405,92) and sublingual misoprostol (560.15 ± 438,00) groups, while the duration of labor was significantly higher in the dinoprostone pessary group (857.37 ± 558,36) (p = 0.000). Hyperstimulation (6.8%) (p = 0.007) and admission rates to the neonatal intensive care unit (9.1%) (p = 0.004) were higher in the dinoprostone group, and this difference was statistically significant.

**Conclusion:**

According to the results of the study, low-dose vaginal or sublingual misoprostol was found to be more effective and safer than vaginal dinoprostone pessary in labor induction.

## Introduction

Labor induction represents one of the most frequently utilized interventions in contemporary obstetric practice, performed through mechanical or pharmacological approaches when maternal or fetal conditions necessitate the initiation of labor [1]. Despite its routine application, the procedure remains clinically challenging due to variation in induction protocols, concerns regarding maternal and neonatal safety, and inconsistencies in reported outcomes. Among pharmacological strategies, prostaglandin-based agents—particularly misoprostol and dinoprostone—constitute the cornerstone of induction regimens, yet their optimal use continues to be debated.

A plethora of medical indications for inducing labor are recommended by international guidelines. These include post-term pregnancy, pre-labor membrane rupture, premature rupture of membranes after 34 weeks, gestational hypertension, gestational diabetes, pre-eclampsia, intrauterine growth retardation, maternal medical reasons, intrauterine fetal death, and so forth [2].

A range of methodologies has been employed to induce cervical ripening, including mechanical techniques such as the use of balloon catheters and amniotomy, as well as pharmacological agents [3]. The Foley catheter is the most commonly used mechanical method [4–6]. The most significant advantages of mechanical methods are that they are inexpensive, simple to store, and readily available. The most commonly used pharmacological agents are misoprostol and dinoprostone.

Misoprostol, a synthetic prostaglandin E1 analog, is widely favored for its low cost, stability at room temperature, and versatility of administration via oral, sublingual, buccal, or vaginal routes [4,6]. However, its administration requires continuous fetal monitoring owing to the risk of uterine hyperstimulation and subsequent fetal compromise [7]. International guidelines offer varying recommendations regarding dosage and intervals, reflecting both the clinical utility of the treatment and the ongoing challenge of achieving standardization [8–10].

Dinoprostone, a synthetic prostaglandin E2 derivative, facilitates cervical ripening, enhances myometrial contractility, and increases oxytocin sensitivity [11]. While effective, it is associated with a 5–15% risk of uterine hyperstimulation and abnormal fetal heart rate tracings, and it cannot be used concomitantly with oxytocin [12]. Furthermore, its requirement for freezer storage and higher cost limit its accessibility in certain clinical settings

Globally, induction rates have increased markedly over the past decades. In the United States, rates rose from 9.6% in 1990 to 27.1% of all births and 37.8% of first births in 2018 [13]. A similar upward trend was observed in Sweden, where the induction rate increased from 13% in 2014 to 25% in 2020 [14].In contrast, data from our country remain limited, underscoring the need for further investigation into induction practices and the effectiveness of pharmacological methods within local clinical contexts.

The present study was therefore designed to retrospectively evaluate the efficacy of misoprostol and dinoprostone for labor induction. By directly comparing these two agents, the study aims to generate context-specific evidence that informs clinical decision-making and contributes to improved maternal and neonatal outcomes.

### Research questions

1. Do vaginal and sublingual misoprostol, compared with vaginal dinoprostone pessary, increase vaginal delivery rates and shorten the induction-to-delivery interval in labor induction?
2. Do different routes of misoprostol administration (vaginal vs. sublingual) have differential effects on delivery outcomes such as cesarean section rate, need for additional induction, and oxytocin augmentation?
3. Are there significant differences in maternal (e.g., hyperstimulation, mode and duration of delivery) and neonatal outcomes (e.g., Apgar scores, birth weight, NICU admission) between the induction methods used in this study?

## Materials and methods

### Study design and setting

This single-center retrospective cohort study included pregnant women who gave birth in a tertiary education and research hospital between April 1, 2021, and April 1, 2023. The study comprised 1,456 pregnant women who received induction for labor during the specified period, had a Gestational age of≥37 weeks, no fetal malformation, a Bishop score below 5, and a live singleton and vertex pregnancy. Patients who could not take misoprostol due to diseases such as epilepsy, asthma, glaucoma, those who were allergic to misoprostol, and those who had uterine surgery such as cesarean section and myomectomy were not included in the study.

### Sample size consideration

Since this was a retrospective cohort study, a formal sample size calculation was not performed. Instead, all eligible cases recorded in the hospital system during the two-year study period were included, which provided a sufficiently large cohort (n = 1,456) for robust statistical analysis. The number of participants in each cohort has been specified**:** vaginal misoprostol group (n = 460), sublingual misoprostol group (n = 455), and dinoprostone pessary group (n = 541).

### Study population

The study data were obtained from the hospital automation system and patient files between March 1, 2024, and April 1, 2024, and recorded in a spreadsheet (Microsoft Excel™) environment. The demographic data encompassed patient age, gravidity, parity, abortion numbers, and Bishop scores. The gestational age of the patients was calculated according to the date of their last menstrual period. The gestational age was calculated using first-trimester crown-rump length (CRL) for patients with unknown last menstrual periods. A total of **1,456 pregnant women** who underwent labor induction during the study period were included.

### Study groups

The study comprised three groups, as determined by the data obtained from the archive scan. The first group comprised pregnant women who were administered 25 μg of vaginal misoprostol for the induction of labor. Misoprostol, the active ingredient in Cytotec®, is available in our country in a 200 μg tablet formulation. In routine practice, 25 μg of misoprostol is administered by way of the posterior fornix of the vagina. If necessary, repeat the dose every six hours, as recommended by ACOG(American College of Obstetricians and Gynecologists) and WHO(World Health Organization), for a maximum of four doses [10,15].

The second group consisted of patients who received 25 μg of sublingual misoprostol for labor induction. Patients receiving sublingual administration are instructed to avoid swallowing the medication and to hold it under the tongue for at least 4–5 minutes. If the first dose is insufficient, the dose is repeated every four hours, as recommended by ACOG and WHO, and a maximum of four doses are administered [10,15].

The third group comprised pregnant women who were administered dinoprostone for labor induction. Dinoprostone is available in our country in the form of a pessary containing 10 mg of dinoprostone, with a release rate of 0,3 mg/hour for 24 hours, under the proprietary name Propess®. The dinoprostone pessary is applied vaginally and positioned in the posterior fornix of the vagina. In the event that the patient does not enter active labor within 24 hours, the dinoprostone pessary is removed. In patients who enter the active phase, the dinoprostone pessary is removed, and induction is continued with a low-dose oxytocin protocol. The oxytocin infusion is initiated 60 minutes after the dinoprostone pessary is removed. The patients did not include those who expelled the pessary prematurely and received additional induction.

### Indications for induction

In the context of clinical practice, irrespective of the induction method, upon the onset of active labor and the attainment of cervical dilation at 6 cm, a low-dose oxytocin protocol is implemented as per the recommendations set forth by the ACOG [10], if deemed necessary for augmentation. According to this protocol, oxytocin is initiated at a rate of 0.5–2 mU/min, and the dosage is increased by 1–2 mU/min every 30 minutes if necessary. Epidural anesthesia is available upon request; however, due to its limited popularity among the local population, it was not utilized in the present study. Apart from the aforementioned treatments, no additional interventions are applied for induction purposes. Patients are monitored in the ward during the initial 24-hour period. Postpartum antibiotics are routinely administered, and analgesics are administered if needed. Patients who will undergo induction for labor are monitored with fetal cardiotocography for one hour before and after induction and every two hours thereafter. Amniotomy is performed when cervical dilation reaches 6 cm.

In all groups, oxytocin infusion is initiated as an additional induction method in the event of induction failure.

The indications for labor induction were determined as follows: post-term gestation (≥287 days), oligohydramnios, gestational hypertension, chronic hypertension, mild pre-eclampsia, gestational or pregestational diabetes, intrauterine growth retardation (IUGR), pregnancy cholestasis, and premature rupture of membranes (PROM), according to the opinion of the Turkish Maternal-Fetal Medicine and Perinatology Association. Patients meeting these criteria were included in the study.

### Outcomes

The primary aim of the study was to evaluate the vaginal delivery rates in the first 24 hours after induction and the time from induction to delivery of the fetus in all groups. The secondary objectives of the study were to determine the rates of cesarean delivery, cesarean indications, first- and fifth-minute Apgar scores, birth weight, gender, amniotic fluid meconium contamination, and admission to the neonatal intensive care unit (NICU).

### Ethics approval

All authors affirm that the research was conducted in accordance with the relevant ethical guidelines and regulations, and in compliance with the principles outlined in the Declaration of Helsinki. The study protocol was reviewed and approved by the Atatürk University Faculty of Medicine Clinical Research Ethics Committee (Approval Date: March 30, 2023; Ref. No: B.30.2.ATA.0.01.00/187). Additional permission was granted by the Scientific Research Permission Committee of the Ağrı Provincial Health Directorate (Letter No. 109, May 10, 2023). As this was a retrospective study based on secondary data obtained from hospital records, the ethics committee waived the requirement for written informed consent.

The authors had temporary access to identifiable data during collection, but all data were anonymized prior to analysis to ensure participant confidentiality.

### Statistical analysis

Data were analyzed using IBM SPSS Statistics version 28.0 (IBM Corp., Armonk, NY, USA). Continuous variables were presented as mean ± standard deviation (SD), and categorical variables as frequencies and percentages. The normality of the data was assessed using the Kolmogorov–Smirnov test and by evaluating skewness–kurtosis values.

For comparisons among groups, One-way ANOVA and post hoc tests (Tukey or Games–Howell) were used for normally distributed variables, while Chi-square or Fisher’s exact tests were used for categorical data. Independent t-tests were applied for pairwise comparisons where appropriate.

A multivariable logistic regression analysis was performed to identify independent predictors of vaginal delivery, adjusting for potential confounders such as maternal age, parity, Bishop score, and gestational week. A p-value <0.05 was considered statistically significant with a 95% confidence interval.

## Results

Table 1 presents the demographic and obstetric characteristics of the patients grouped by category. Women in the dinoprostone group were generally younger compared with those in both misoprostol groups. Gestational age at delivery was similar across the three groups. With respect to obstetric history, women in the dinoprostone group had lower gravidity and parity compared with their counterparts in the vaginal and sublingual misoprostol groups. Likewise, a history of abortion was less common among women in the dinoprostone cohort. Regarding cervical status, the distribution of Bishop scores was similar across all three groups, with approximately two-thirds of patients presenting with a score between 1–3, and about one-third presenting with a score of 4. Taken together, these findings indicate that patients in the dinoprostone group tended to be younger and had less extensive reproductive histories, while gestational age and cervical readiness (Bishop score) were comparable across all groups.

**Table 1:** Distribution of demographic and obstetric characteristics by groups.

Variables		Vaginal Misoprostol (a) N = 460	Sublingual Misoprostol (b) N = 455	Dinoprostone Pessary (c) N = 541	Test Value	P-Value
		X ± SD	X ± SD	X ± SD		
**Maternal Age**		27.75 ± 6.12	27.25 ± 5.54	25.17 ± 4.62	F = 32.352 **c < a, b < a**	0.000**
**Gestational age**		39.77 ± 1.39	39.80 ± 1.32	39.68 ± 1.41	F = 0.988	0.373*
**Gravida**		3.08 ± 1.98	2.85 ± 2.07	1.75 ± 1.18	F = 82.949 **c < b, c < a**	0.000**
**Parity**		1.63 ± 1.64	1.49 ± 1.71	0.47 ± 0.92	F = 96.941 **c < b, c < a**	0.000**
**History of Abortion**		0.46 ± 0.88	0.36 ± 0.85	0.28 ± 0.59	F = 6.809 **c < a, c < b**	0.001**
**Bishop score**	**(1 –3 )**	300(65.2)	291(64.0)	353(65.2)	χ²(2) = 0.224	0.894***
	**4**	160(34.8)	164(36.0)	188(34.8)		

*One-Way ANOVA,

**POST HOCT TESTES (BONFERRONİ), (It has been applied to control the Type I error rate in multiple comparisons)

****Pearson Chi-Square

The distribution of hospitalization indications across the induction groups is presented in Table 2. Statistically significant differences were observed among the groups (*p* < 0.001). Overall, the most frequent indications were oligohydramnios (29.6%), term pregnancy (29.6%), PROM (22.7%), and IUGR (7.0%). PROM was predominantly observed in the misoprostol groups (28.9% in the vaginal and 31.0% in the sublingual group), whereas it was markedly lower in the dinoprostone group (10.5%). In contrast, oligohydramnios (34.4%) and IUGR (10.4%) were more common in the dinoprostone group compared with the misoprostol group (approximately 26–28% and 4–6%, respectively). Term pregnancy was comparably distributed across all groups (26.6–32.0%). Other maternal indications, including gestational hypertension (0.9–3.5%), post-term pregnancy (7.0–7.9%), gestational diabetes (0.2–0.4%), and intrahepatic cholestasis of pregnancy (0.4–1.1%), were infrequent and did not differ substantially between groups.

**Table 2:** Distribution of hospitalization ındications by groups.

Variables		Vaginal Misoprostol (a) N = 460	Sublingual Misoprostol (b) N = 455	Dinoprostone Pessary (c) N = 541	Test Value	P-Value
		N (%)	N (%)	N (%)		
**Indication for Hospitalization**	**PROM**	133 (28.9)	141 (31.0)	57 (10.5)	**X² = 102.465**	0.000*
	**Oligohydramnios**	119 (25.9)	126 (27.7)	186 (34.4)		
	**Term Pregnancy**	137 (29.8)	121 (26.6)	173 (32.0)		
	**IUGR**	20 (4.3)	26 (5.7)	56 (10.4)		
	**Gestational Hypertension**	15 (3.3)	4 (0.9)	19 (3.5)		
	**Post-term pregnancy**	32 (7.0)	34 (7.5)	43 (7.9)		
	**Gestational Diabetes**	2 (0.4)	1 (0.2)	1 (0.2)		
	**Cholestasis of Pregnancy**	2 (0.4)	2 (0.4)	6 (1.1)		

*Pearson Chi-Square

Table 3 illustrates a comparative analysis of maternal outcomes by group, revealing notable findings. When the groups were compared according to the types of delivery, vaginal delivery rates were observed to be similar in both the misoprostol groups and higher than the dinoprostone pessary group. Conversely, a higher incidence of cesarean sections was observed in the dinoprostone pessary group, a difference that proved statistically significant (p = 0.000). No statistically significant difference was found between the groups regarding cesarean section indications (p = 0.225). A closer look at the cesarean indications reveals that the most prevalent reasons for cesarean section in all three groups were fetal distress and non-progressive labor.

**Table 3:** Distribution of maternal outcomes by groups.

Variables		Vaginal Misoprostol N = 460	Sublingual Misoprostol N = 455	Dinoprostone Pessary N = 541	Test Value	P-Value
		N (%)	N (%)	N (%)		
**Type of Birth**	**Caesarean Birth**	62 (13.5)	67 (14.7)	171 (31.6)	**X² = 63.935**	0.000*
	**Vaginal Birth**	398 (86.5)	388 (85.3)	370 (68.4)		
**Indication for Caesarean section**	**Fetal distress**	42 (67.7)	48 (71.6)	108 (63.2)	X² = 12.985	0.225*
	**Non-progressive action**	15 (24.2)	16 (23.9)	52 (30.4)		
	**Cord prolapse**	0 (0.0)	1 (1.5)	1 (0.6)		
	**Placental Abruption**	3 (4.8)	2 (3.0)	7 (4.1)		
	**Maternal incompatibility**	2 (3.2)	0 (0.0)	0 (0.0)		
	**Severe Preeclampsia**	0 (0.0)	0 (0.0)	3 (1.8)		
**Need for additional induction**	**Yes**	18 (3.9)	23 (5.1)	106 (19.6)	**X² = 85.873**	0.000*
	**No**	442 (96.1)	432 (94.9)	435 (80.4)		
**Delivery without Oxytocin**	**Yes**	355 (40.3)	331 (37.6)	195 (22.1)	**X² = 217.485**	0.000*
	**No**	105 (18.3)	124 (21.6)	346 (60.2)		
**Hyperstimulation/Tachysystole**	**Yes**	15 (3.3)	15 (3.3)	37 (6.8)	**X² = 9.819**	0.007*
	**No**	445 (96.7)	440 (96.7)	504 (93.2)		
		**X ± SD**	**X ± SD**	**X ± SD**		
**Time to Delivery (min)**	**Total**	**523.86 ± 405.92**	**560.15 ± 438.00**	**857.37 ± 558.36**	**F = 74.903**	0.000**
	**Primpar**	**649.83 ± 516.27**	**665.05 ± 532.11**	**935.97 ± 557.32**	**F = 23.968** c > a,c > b***	0.000**
	**Multipara**	**461.70 ± 321.75**	**487.61 ± 341.49**	**638.60 ± 501.70**	**F = 11.648** c > a,c > b***	0.000**
**Number of misoprostol doses**		1.25 ± 0.66	1.30 ± 0.69	–	F = 0.977	0.323

*Pearson Chi-Square **

**One-Way ANOVA,

***POST HOCT TESTES (BONFERRONİ), (It has been applied to control the Type I error rate in multiple comparisons)

The groups were compared according to the need for additional induction. No statistically significant difference was observed between the vaginal and sublingual misoprostol groups in terms of the need for additional induction. Conversely, the requirement for additional induction was found to be statistically significantly higher in the dinoprostone group (p = 0.000).

We compared patients who delivered without requiring oxytocin induction during the active phase across the three groups. The number of patients who gave birth without requiring oxytocin induction was higher in both misoprostol groups than in the dinoprostone group, and these results were statistically significant (p = 0.000).

The groups were compared according to the hyperstimulation status that developed after the induction application. Hyperstimulation was detected at a statistically significantly higher rate in the dinoprostone group (p = 0.007).

The groups were compared according to the duration of labor. While the duration of labor in the vaginal and sublingual misoprostol groups exhibited similarity, the duration of labor in the dinoprostone group was found to be prolonged (p = 0.000). A further comparison was made between the groups regarding the duration of labor, specifically for primiparous and multiparous women. In both instances, the duration of labor was found to be significantly longer in the dinoprostone group (p = 0.000).

The vaginal and sublingual misoprostol groups were analyzed for the mean misoprostol dose, and no statistically significant difference was found between the groups (p = 0.323).

The fetal outcomes were compared by groups in Table 4. To assess the well-being of the newborns, groups were compared based on their first- and fifth-minute Apgar scores. The investigation revealed no statistically significant difference between the groups with regard to either the first-minute (p = 0.082) or fifth-minute (p = 0.087) Apgar scores.

**Table 4:** Distribution of fetal outcomes by groups.

Variables		Vaginal Misoprostol (a) N = 460	Sublingual Misoprostol (b) N = 455	Dinoprostone Pessary (c) N = 541	Test Value	P-Value
		X ± SD	X ± SD	X ± SD		
**1st minute Apgar**		7.91 ± 0.39	7.83 ± 0.63	7.85 ± 0.57	F = 2.504	0.082*
**5th minute Apgar**		8.96 ± 0.22	8.90 ± 0.52	8.92 ± 0.43	F = 2.445	0.087*
**Newborn Weight**		3157.98 ± 394,61	3157.21 ± 399.746	3070.79 ± 441,65	**F = 7.464** **c < b = a**	0.001**
		**n (%)**	**n (%)**	**n (%)**		
**Gender**	**Girl**	197 (42.8)	218 (47.9)	268 (49.5)	X² = 4.764	0.092 ***
	**Male**	263 (57.2)	237 (52.1)	273 (50.5)		
**NICU Admission**	**Yes**	18 (3.9)	37 (8.1)	49 (9.1)	**X² = 10.895**	0.004***
	**No**	442 (96.1)	418 (91.9)	492 (90.9)		
**Meconium**	**Yes**	58 (12.6)	61 (13.4)	65 (12.0)	X² = 0.434	0.805 ***
	**No**	402 (87.4)	394 (86.6)	476 (88.0)		

*One-Way ANOVA Test,

**POST HOCT TESTES (BONFERRONİ), (It has been applied to control the Type I error rate in multiple comparisons)

***Pearson Chi-Square

A subsequent comparison of the groups, based on the weights of the newborns, revealed that the newborns in the dinoprostone group had a lower mean weight compared to the control group (p = 0.001). This finding was deemed to be statistically significant.

There was no significant difference between the groups in terms of the gender of the newborns (p = 0.092).

A comparison was made between the groups regarding admission rates to the NICU. The lowest rate of NICU admission was observed in the group administered vaginal misoprostol. The results were similarly elevated in the group administered sublingual misoprostol and dinoprostone, which were statistically significant (p = 0.004).

In conclusion, a comparison was made between the groups according to the status of meconium transmission in amniotic fluid. No significant difference was found between the groups regarding meconium transmission in amniotic fluid. The transmission of meconium in amniotic fluid was found to be statistically similar in all three groups (p = 0.805).

Binary logistic regression analysis with vaginal birth as the dependent variable yielded a statistically significant model (χ² (8) = 10.070, *p* < 0.001), which demonstrated an adequate fit (Hosmer–Lemeshow test, *p* = 0.260) and explained approximately 18% of the variance in vaginal birth (Nagelkerke R² = 0.184).

Maternal age was negatively associated with the likelihood of vaginal delivery (OR = 0.95, 95% CI: 0.92–0.98, *p* = .003), indicating that increasing maternal age was an independent risk factor for cesarean birth. Conversely, parity was positively associated with vaginal delivery (OR = 1.39, 95% CI: 1.21–1.59, *p* < .001), suggesting that multiparous women were significantly more likely to deliver vaginally. Gestational week showed a borderline effect (OR = 1.09, 95% CI: 0.99–1.21, *p* = .075). Importantly, Bishop score emerged as a strong determinant of vaginal delivery (OR = 1.38, 95% CI: 1.15–1.65, *p* < .001).

Regarding pharmacological induction methods, both vaginal misoprostol (OR = 0.45, 95% CI: 0.31–0.64, *p* < .001) and sublingual misoprostol (OR = 0.47, 95% CI: 0.33–0.66, *p* < .001) were significantly associated with higher odds of vaginal delivery compared to women who did not receive these agents. The presence of meconium in the amniotic fluid was found to be the strongest predictor in the model: women without meconium had a four-fold greater likelihood of achieving vaginal birth compared to those with meconium (OR = 4.01, 95% CI: 2.82–5.71, *p* < .001). Duration of labor exerted a statistically significant but clinically negligible effect (OR = 1.000, 95% CI: 0.999–1.000, *p* < .001) (Table 5)

**Table 5:** Results of logistic regression analysis of factors influencing vaginal birth.

		95% C.I.for EXP(B)				
Variables	aOR	Lower	Upper	p	R^2^	HLp
Maternal age	.954	.924	.984	.003	0.184	0.260
Parity	1.385	1.206	1.590	.000		
Bishop score	1.378	1.153	1.646	.000		
Gestational age(week)	1.093	.991	1.206	.075		
Delivery time	1.000	.999	1.000	.000		
Meconium	4.009	2.815	5.709	.000		
Vaginal misoprostol	.450	.314	.644	.000		
Sublingual misoprostol	.467	.330	.661	.000		

Dependent variable:. vaginal birth yes/no; OR: Odds ratio; HLp: Hosmer and Lemeshow.

Taken together, these findings indicate that younger maternal age, higher parity, advanced Bishop score, and the absence of meconium were the most influential predictors of successful vaginal delivery. Moreover, both vaginal and sublingual misoprostol were effective in improving the likelihood of vaginal birth, supporting their clinical utility as induction agents.

## Discussion

The present study demonstrated that both vaginal and sublingual misoprostol significantly enhanced the likelihood of vaginal delivery compared with dinoprostone, even after adjusting for key confounders such as maternal age, parity, Bishop score, and gestational age. Our multivariable logistic regression analysis showed that misoprostol nearly doubled the probability of vaginal birth, underscoring its robust clinical efficacy. These findings are consistent with prior large-scale systematic reviews and network meta-analyses that have identified misoprostol as one of the most effective pharmacological agents for labor induction [3,7]. Furthermore, international guidelines, including those from FIGO (International Federation of Gynecology and Obstetrics) and the WHO, endorse the use of low-dose misoprostol (25 µg) as both a safe and effective method of induction [9,15].

Swift et al. conducted a population-based study in Iceland, investigating the indications for inducing patients who gave birth between 1997 and 2018. The study’s findings indicated that the predominant indications for induction of labor included prolonged pregnancy, hypertensive diseases, and gestational diabetes [16]. Coates et al. conducted a systematic review of the indications for labor induction and identified the four most common: post-term pregnancy, gestational hypertension/pre-eclampsia, diabetes, and pre-labor rupture of membranes (PROM) [17]. In a study conducted by Mayer et al., it was reported that the three most prevalent indications for induction were prolonged pregnancy, PROM, and intrauterine growth restriction (IUGR) [18]. In the present study, the most prevalent indications for labor induction were identified as follows: PROM, oligohydramnios, term pregnancy, and intrauterine growth retardation.

In their meta-analysis, Ramadan and colleagues examined 53 randomized controlled trials. The study’s findings indicated that vaginal misoprostol exhibited superior efficacy in inducing labor when compared with dinoprostone. The analysis revealed that the vaginal delivery rate within 24 hours was higher in the group administered with misoprostol. Furthermore, the study revealed that the necessity for oxytocin augmentation was reduced in the vaginal misoprostol group compared to the dinoprostone group. However, no significant differences were observed between oral misoprostol and dinoprostone. Furthermore, no significant differences were observed in cesarean delivery rates between the vaginal misoprostol and dinoprostone groups [19]. In their meta-analysis, Zheng and colleagues compared the use of misoprostol and dinoprostone. The analysis revealed no significant disparities between the two groups with respect to the rates of vaginal and cesarean delivery. However, they observed that the dinoprostone group had a higher requirement for oxytocin augmentation [20]. In their study, Deepika and her colleagues also made a comparison between sublingual misoprostol and intracervical dinoprostone gel. The results of the study indicated that vaginal delivery rates were higher in the sublingual misoprostol group; however, this difference was not statistically significant [21]. In their systematic review and meta-analysis, Ma and Chen compared vaginal misoprostol with intracervical dinoprostone. The analysis revealed that the vaginal delivery rate within 24 hours was higher in the group administered vaginal misoprostol. However, there was no significant difference in cesarean delivery rates between the two groups [22]. Maggi et al. conducted a retrospective study of 220 patients who had undergone misoprostol vaginal insert (MVI) and dinoprostone vaginal insert (DVI). The study revealed that the MVI group exhibited higher vaginal birth rates, while the DVI group demonstrated higher cesarean birth rates [23]. In a retrospective study, Mayer et al. also made a comparison between MVI and DVI. The study revealed that the rates of vaginal and cesarean births were similar in both groups [18]. In a subsequent study, Veena and colleagues compared sublingual misoprostol with vaginal PGE2 gel. The results of this study indicated that there were higher vaginal birth rates and lower cesarean birth rates in the sublingual misoprostol group [24]. In a randomized controlled trial, Wing and colleagues compared MVI and DVI. The study revealed that the rates of vaginal and cesarean births were similar between the two groups [25]. In the present study, vaginal delivery rates were found to be statistically similar in both misoprostol groups. Conversely, the cesarean delivery rate was higher in the dinoprostone group than in the other groups. Furthermore, a greater number of patients in the misoprostol group gave birth without requiring oxytocin during the active phase when compared to those in the dinoprostone pessary group. Conversely, the dinoprostone pessary group exhibited a higher incidence of patients requiring additional induction. When the cesarean indications were examined, the most common in all three groups were fetal distress and non-progressive labor.

In their respective studies, Deepika et al. [21] and Veena et al. [24] evaluated the duration of labor by comparing sublingual misoprostol with dinoprostone gel. The findings of these studies indicated that the duration of labor was shorter in the misoprostol group, a finding consistent with the results of the present study. In studies comparing MVI with DVI, the durations from induction to delivery were reported to be shorter in the MVI group [23,25]. In addition, Rhadika and colleagues compared vaginal misoprostol with intracervical dinoprostone and found shorter labor times in the dinoprostone group [26]. In the present study, the time from induction to delivery was similar in both misoprostol groups, whereas it was longer in the dioprostone group. In our study, the number of nulliparous patients was higher in the dinoprostone group. To prevent this from affecting the results, we compared the groups by separating them into nulliparous and multiparous categories, and the results remained the same. In the dinoprostone group, the time from induction to delivery was longer in all groups.

Hyperstimulation is defined as the occurrence of five or more uterine contractions within a 10-minute period, accompanied by a deterioration in fetal cardiac activity [27]. The increase in the incidence of tachysystole or hyperstimulation in pregnant women who were administered misoprostol is a result emphasized in many studies [28–30]. Maggi et al. also reported a higher rate of uterine tachysystole in the MVI group in their study [23]. In a randomized controlled trial, Jahromi et al. compared the use of vaginal and sublingual misoprostol for the induction of labor in term pregnant women. The study’s findings indicated that the incidence of hyperstimulation was twice as high in the sublingual group [31]. In a comparative study, Feitoza et al. examined the utilization of vaginal and sublingual misoprostol for the induction of labor, observing comparable rates of hyperstimulation in both groups [32]. Taliento et al. found no difference in hyperstimulation between vaginal misoprostol and dinoprostone pessary in their systematic review and meta-analysis. However, in subgroup analyses of studies that exclusively compared oral misoprostol and dinoprostone pessaries, the researchers observed a higher incidence of hyperstimulation in the dinoprostone group. In a further analysis of studies comparing vaginal misoprostol and dinoprostone gel, it was reported that hyperstimulation was higher in the vaginal misoprostol group [33]. In their systematic review and meta-analysis study, Ma and Chen also compared the use of vaginal misoprostol with intracervical dinoprostone. The study found higher rates of hyperstimulation/tachysystole in the misoprostol group [22]. Contrary to the findings of numerous studies in the extant literature, our study demonstrated similar rates of hyperstimulation in both misoprostol groups. Furthermore, a higher rate of hyperstimulation was observed in the dinoprostone pessary group compared to the other two groups.

In their systematic review and meta-analysis, Taliento et al. found no significant difference between misoprostol and dinoprostone regarding fifth-minute Apgar scores [33]. In their study comparing vaginal misoprostol with DVI, Bennet et al. found no difference in Apgar scores between the groups [34]. Zheng et al. also compared vaginal misoprostol with DVI in their meta-analysis study. The study results showed no difference between the groups in terms of Apgar scores and admissions to the NICU unit [20]. Ma and Chen also found no difference in Apgar scores and admission to the NICU unit between the vaginal misoprostol and intracervical dinoprostone groups in their study [22]. In the present study, no significant differences were observed between the groups with regard to Apgar scores. However, when the rate of hospitalization to the NICU was examined, it was found that the sublingual misoprostol and dinoprostone pessary group exhibited a higher rate of hospitalization to the NICU compared to the vaginal misoprostol group. This result was found to be statistically significant. Although the difference in NICU admission rates between the groups reached statistical significance, it is unlikely to be of clinical relevance. No concurrent increases were observed in adverse neonatal outcomes such as low Apgar scores, asphyxia, or neonatal mortality. Most NICU admissions were precautionary, for short-term observation or transient respiratory monitoring, rather than due to serious neonatal complications. Therefore, the observed difference may reflect institutional monitoring practices rather than a true increase in neonatal morbidity.

In their systematic review and meta-analysis comparing misoprostol and dinoprostone, Taliento et al. found no difference in the incidence of meconium-stained amniotic fluid between the groups [33]. Ramadan and his colleagues also compared misoprostol and dinoprostone in their study. As a result of the study, they found higher rates of amniotic fluid meconium staining in the vaginal misoprostol group [19]. Chitrakar compared vaginal misoprostol with intracervical dinoprostone in his research and found a higher rate of meconium-stained amniotic fluid in the dinoprostone group. Still, this result was not statistically significant [35].

A potential limitation of this study is the difference in administration intervals between the vaginal and sublingual misoprostol groups (6 hours vs. 4 hours). Although both regimens were low-dose and guideline-based, the observed differences in outcomes may reflect not only the route of administration but also the dosing schedule. This should be taken into account when evaluating the comparative effectiveness of the two approaches.

From a clinical standpoint, the findings of this study reinforce the role of low-dose misoprostol as a safe, effective, and practical first-line agent for labor induction. Both vaginal and sublingual routes demonstrated comparable efficacy, supporting their use in individualized clinical decision-making based on patient comfort, provider experience, and institutional protocols. The shorter induction-to-delivery interval and reduced need for oxytocin augmentation observed with misoprostol may translate into improved labor efficiency and resource utilization, particularly in settings with limited clinical capacity. Future research should focus on prospective, multicenter randomized controlled trials to validate these results and establish optimal dosing intervals between different routes of administration. Furthermore, incorporating patient-reported outcomes and neonatal long-term follow-up data would enhance understanding of the overall safety profile and satisfaction associated with pharmacological induction strategies.

## Conclusion

The findings of this study demonstrate that low-dose (25 µg) misoprostol is a safe and effective option for labor induction. Although previous reports have suggested that dinoprostone may be more successful and associated with fewer adverse events, while misoprostol has been linked to hyperstimulation, fetal distress, and meconium passage, our results indicate that misoprostol achieved higher induction success compared with dinoprostone. The adverse effects of misoprostol appear to be more pronounced with higher doses, whereas the low-dose regimen employed in this study was associated with favorable maternal and neonatal outcomes.

When routes of administration are considered, vaginal misoprostol exerts a strong local effect on the cervix but requires provider involvement and may limit patient comfort. In contrast, sublingual misoprostol offers rapid absorption, ease of administration, and greater acceptability for both patients and healthcare staff, making it a more practical and non-invasive approach. Therefore, in clinical practice, sublingual misoprostol may be preferred, particularly in settings where patient autonomy and convenience are prioritized. Nevertheless, further large, multicenter randomized controlled trials are warranted to determine the optimal route of administration and confirm long-term safety.

### Strengths and limitations

The present study acknowledges both its strengths and limitations. A primary limitation is its retrospective and single-center design, which may restrict the generalizability of the findings to broader populations. However, the study also possesses several strengths, including the use of standardized induction protocols**,** well-defined inclusion and exclusion criteria, the homogeneity of the study population, and a large sample size, all of which contribute to the internal validity and reliability of the results.

## Abbreviations

WHO: World Health Organization
FIGO: International Federation of Gynecology and Obstetrics
ACOG: American College of Obstetrics and Gynecology
CRL: Crown-rump length
NICU: Neonatal Intensive Care Unit
MVI: Misoprostol Vaginal Insert
DVI: Dinoprostone Vaginal Insert.
